# Sepsis and cirrhosis in growing animals: description of a new experimental model and its pathological and immunological reliability

**DOI:** 10.6061/clinics/2020/e1858

**Published:** 2020-09-23

**Authors:** Pedro Augusto Dantas de Moraes, Ana Cristina Aoun Tannuri, Livio Moreira Rios, Vitor Ribeiro Paes, Josiane de Oliveira Gonçalves, Suellen Serafini, Uenis Tannuri

**Affiliations:** Divisao de Cirurgia Pediatrica, Unidade Pediatrica de Transplante de Figado e Laboratorio de Pesquisa em Cirurgia Pediatrica (LIM 30), Faculdade de Medicina FMUSP, Universidade de Sao Paulo, Sao Paulo, SP, BR

**Keywords:** Cirrhosis, Immune Dysfunction, Endotoxemia, Experimental Cirrhosis, Biliary Obstruction, Biliary Cirrhosis

## Abstract

**OBJECTIVES:**

In cirrhotic children, infection events and sepsis are more frequent and more severe due to immune dysfunction. The objectives of the current study were therefore to develop an experimental model of infection and sepsis in cirrhotic weaning growing rats, by the use of bile duct ligation (BDL) and cecal ligation and puncture (CLP). Additionally, the correlation of the clinico-histopathological data and serial cytokine levels in septic cirrhotic and non-cirrhotic animals was studied.

**METHODS:**

Young Wistar rats of age 21 days and of weight between 70-90 g were divided into 12 groups according to the surgical procedure performed: sham (sacrificed after 2 or 4 weeks), BDL (sacrificed after 2 or 4 weeks), CLP (2- or 4-week old animals sacrificed after 12 or 24 hours), BDL+CLP (2- or 4-week old animals sacrificed after 12 hours). Histopathological studies and determination of serum levels of cytokines IL-1 beta, IL-10, and TNF-alpha, for studies of systemic infection, were performed. Murine sepsis scores (MSS) based on the clinical aspects just before euthanasia were also included.

**RESULTS:**

A transitory increase in IL-1, IL-10, and TNF-alpha levels was observed, with different patterns according to the groups. Two-hit groups tended to present with higher values of serum cytokines and histopathological scores than their septic non-cirrhotic counterparts. There was a correlation between mortality rate and MSS (*p*<0.0001).

**CONCLUSION:**

The model is feasible and may be utilized in studies on liver cirrhosis and infection in growing animals.

## INTRODUCTION

Biliary cirrhosis in childhood, mainly caused by biliary atresia, leads to considerable morbimortality and is the leading indication of liver transplantation in children (1).

In cirrhotic children, infection events and sepsis are more frequent and more severe, being one of the leading causes of death in this population. Immune dysfunction in cirrhotic patients has been classically described (2). Sepsis is a systemic inflammatory response associated with various infectious agents and worsened by different underlying conditions (3). In this scenario, infectious agents are responsible for the activation of mononuclear cells, endothelial cells, polymorphonuclear cells, monocytes, and macrophages. The cellular response involves inflammatory pathways and biomarkers like tumor necrosis factor alpha (TNF-α) and interleukins (ILs) such as IL-1 and IL-10. The accumulation of successive damage would hence result in the progression to greater organic impairments. Grading of the disease according to severity, like systemic inflammatory response syndrome (SIRS), sepsis, severe sepsis, septic shock has been proposed as the clinical evaluation of patients (3). In clinical practice, it is observed that some organs are particularly affected by systemic repercussions of sepsis, like lungs, kidneys, and heart.

Among the experimental models of cirrhosis, common bile duct ligation (BDL) is considered a simple and reliable extrahepatic cholestasis model (4-6). Peritonitis models, induced by cecal ligation and puncture (CLP), are practical and well resemble the human disease (7). The objectives of the current study were therefore to develop an experimental model of infection and sepsis in cirrhotic young animal, by the use of BDL and CLP in weaning growing rats. Additionally, the correlations between clinico-histopathological data, and serial cytokine levels in septic cirrhotic and non-cirrhotic animals were studied.

## METHODS

### Animals

Young Wistar rats of both sexes with age 21 days and weight between 70-90 g were used. The animals were cared for according to the criteria outlined in the “Guide for Care and Use of Laboratory Animals” published by the National Academy of Sciences. The study protocol was reviewed and approved by the Animal Ethics Committee at our institution (University of São Paulo Medical School, São Paulo, Brazil).

**Figure 1:**
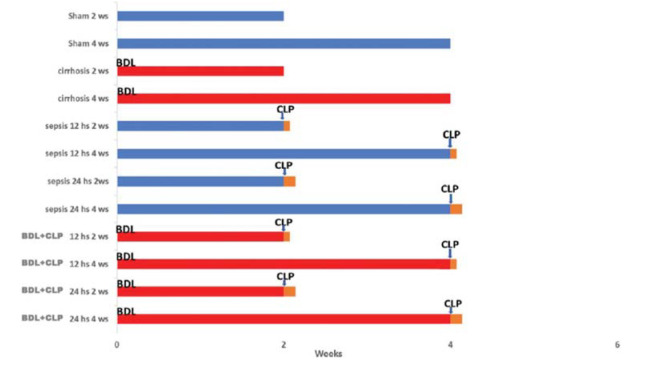
Animal distributions per group.

The experimental animals were divided into groups, as shown in Figure 1. All 12 euthanized groups, each with eight animals, were analyzed at two timepoints, weeks 2 or 4. In the BDL+CLP groups, induction of cirrhosis by BDL and that of sepsis by CLP were performed at either weeks 2 or 4. The sham animals were anesthetized and subjected only to open-and-close laparotomy without BDL, and euthanasia of the CLP groups was performed 12 or 24 hours after the procedure.

### Surgical procedures

The experimental surgical procedures were performed under the supervision of the researcher in charge. The animals were anesthetized with isoflurane (Isothane^®^) by inhalation until sedation, followed by intramuscular doses of 75 mg/kg ketamine hydrochloride (Ketalar^®^).

**BDL:** The anesthetized animals were placed on the procedure table with their legs fixed onto it with adhesive plasters. A median incision of approximately 3 cm was made in the abdomen, just below the xiphoid appendix. The liver and intestines were exteriorized from the abdominal cavity to permit visualization of the bile ducts. Once identified, the common bile duct was dissected, ligated at two points (5 mm apart) with 7.0 prolene suture, and then sectioned between the ligatures. In the sham groups, only an open-and-close laparotomy was performed.

**CLP:** A median laparotomy of 2 cm was performed, and the cecum was exposed. It was ligated closely and distally to the ileocecal valve by 0.5 cm, and was then punctured once with a 21 G needle and tightened to remove the contents. Finally, the viscera was repositioned and the abdominal cavity was closed by a continuous 4.0 prolene single suture.

### Sample collection and preparation

The rats either died naturally or were euthanized by isoflurane inhalation until cardiorespiratory arrest. Cardiac puncture was employed as the blood collection technique. The blood samples were left on ice to avoid hemolysis. A wide laparotomy was used to harvest the liver and kidneys, and a sternotomy was then performed to surgically expose and cannulate the trachea with a 22 G catheter. Heart and lung samples were subsequently collected for histological analysis. The lungs were inflated and fixed at 23 cm pressure with 4% paraformaldehyde in phosphate-buffered solution.

### Histopathology

The tissues were immersed in 10% formaldehyde solution for 24 hours. After the fixation period, they were subjected to dehydration and inclusion in paraffin blocks, which were then sliced to 4-μm thick sections. Histological sections of the heart, lung, kidney, and liver were obtained and the parameters studied were rated from 0 to 3 (0: absence of findings; 1: discreet findings; 2: moderate findings; 3: intense findings) (Table 1). The sums of all scores for each organ were used for comparison.

The analyses were performed by optical microscopy with the aid of the Image Analyzer system. The workstation consisted of a Zeiss Binocular Light microscope, an image digitizing board, and a microcomputer with a Pentium processor, Windows^©^. The images were obtained with the help of Image Pro Plus software version 4.5 (Media Cybernetics^©^), specific for image analysis so that the different variables were quantified and the results transferred to a spreadsheet (Microsoft Excel^©^). The histological sections, in turn, were analyzed using 10 photos of each slide acquired at a magnification of 200x (20x objective and 10x eyepiece). All measurements were archived in a computer-specific program (SPSS-15.0, SPSS inc^©^) for subsequent statistical analysis.

### Interleukins

The interleukins of choice were IL-1 beta, IL-10, and TNF-alpha, which are very commonly used in studies of systemic infections. Blood samples were first collected at a sufficient amount, and were conditioned in dry tubes to maintain stability of serum cytokines. Subsequently, the samples were coagulated at room temperature for 30-45 minutes, and centrifuged at 4°C at a speed of 4,000 rpm for 20 minutes. From the resultant serum, a 30 µL aliquot was separated and conditioned at a temperature of -70°C while avoiding thawing.

Samples were then prepared for analysis on 96-well microplates using a custom Milliplex MAP-type panel. The Cytokine Chemokine Magnetic Bead Panel (Milipore Corp., Billerica, MA) was performed following the specific Millipore protocols. The analytes were quantified using the Magpix analytical testing tool which uses xMAP^TM^ technology (Luminex Corp., Austin, TX) as well as xPONENT 4.2 software (Luminex^®^).

Cytokine concentrations (pg/mL) were determined based on the adjustment of a standard curve for mean fluorescence intensity *versus* pg/mL (or ng/mL).

### Scores

As in human medicine, scores given based on a set of observable clinical criteria are important when it comes to the reproducibility of experimental models; each model may have distinct sensitivities and specificities depending on the pathology and outcome. We therefore utilized the murine sepsis score (MSS) based on analysis of clinical aspects just before euthanasia (8) (Table 2).

### Statistics

Data were stored in excel files and analyzed with R Commander, SPSS and GraphPad Prism. For the initial interpretation of data, distribution type was verified through the Shapiro-Wilk test in order to evaluate most suitable test for each comparison.

Data that presented a normal distribution (parametric) were analyzed using the student's T-test (for two variables) or ANOVA (for more than two variables), followed by the Tukey test to verify the statistical difference between the groups. If the distribution was not normal, the data were evaluated using the non-parametric Mann-Whitney test (for two variables) or Kruskal-Wallis test (for more than two variables), followed by the determination of significant differences between groups using the Dunn test.

Correlation of MSS values with laboratory results, histological parameters, and mortality rates were performed using the Spearman correlation coefficient.

Categorical data were expressed as percentages while continuous variables as mean ± standard deviation. Ordinal measures were expressed as median and quartiles.

The equality hypothesis was rejected if *p*≤0.05.

## RESULTS

A total of 118 rats were used in the experiment, with a constant number of eight animals per group. The mortality distribution per group is indicated in Table 3:

### Cytokines

**Figure 2:**
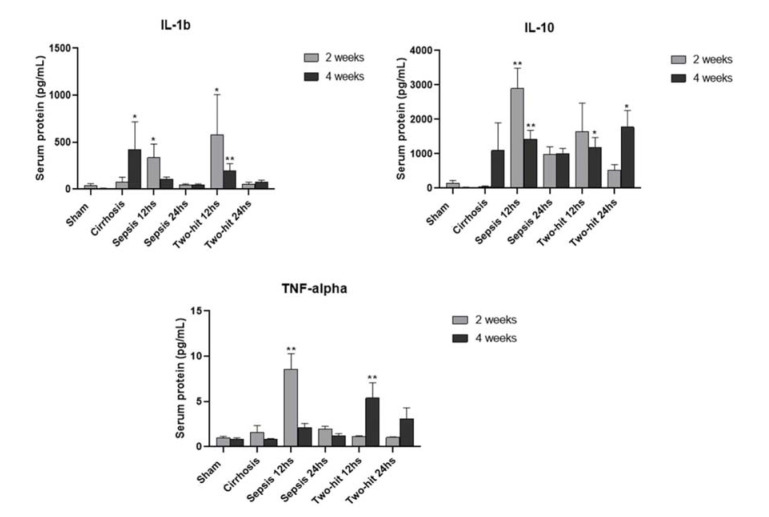
Cytokine levels in the experimental groups (mean serum protein concentrations ± SEM). *p*<0.0001 for comparison between all groups. *p<0.05, **p<0.01 for comparisons against the sham group (respective week).

The results of cytokine levels are shown in Figure 2. It was verified that the groups of rats tended to have higher levels of serum cytokines at week 2. Groups with 12-hour observation tended to present higher values for IL-1 beta and IL-10 than the 24-hour groups. TNF-alpha had increased values at week 2, and the BDL+CLP groups presented higher values than their septic counterparts. There was a significant statistical difference (*p*<0.001) when comparing the groups in general, however, a 2-to-2 comparison showed no significant data, except for the comparison between the experimental and the control groups. The sham groups did not show any significant difference at both weeks 2 and 4.

### Histopathology

**Figure 3:**
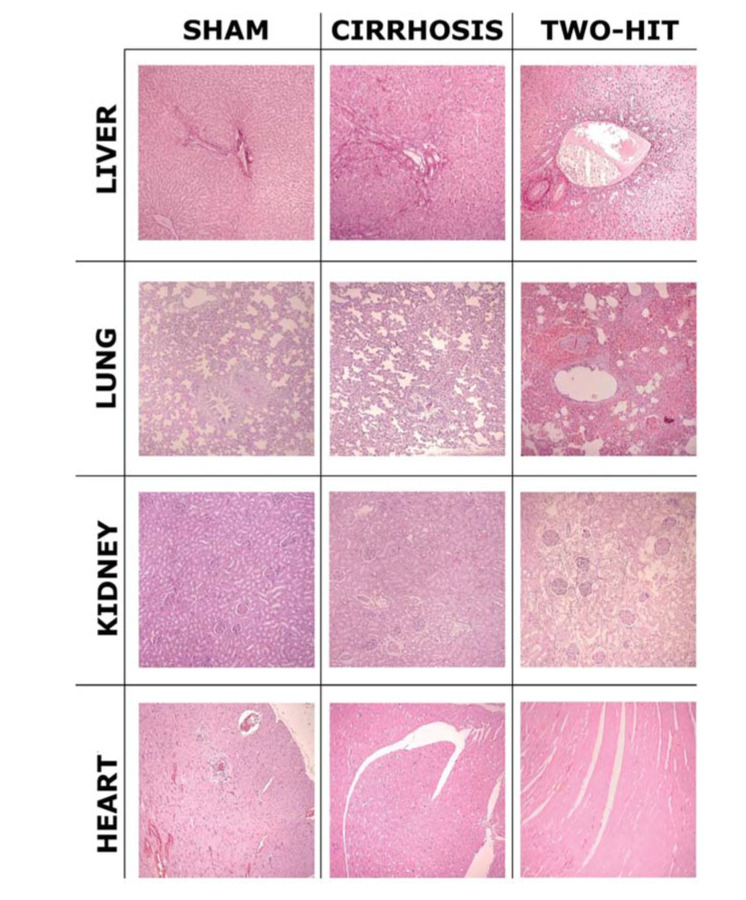
Main histological alterations observed in the experimental animals. In the liver, note that the portal tract is expanded because of intense ductular proliferation. In the lung, note the reduced alveolar space and thickening of pulmonary septa in the BDL+CLP animals. Observe the aspect of renal parenchyma edema in the two-hit animal. No alterations were observed in heart histology. (magnification 100X).

**Figure 4:**
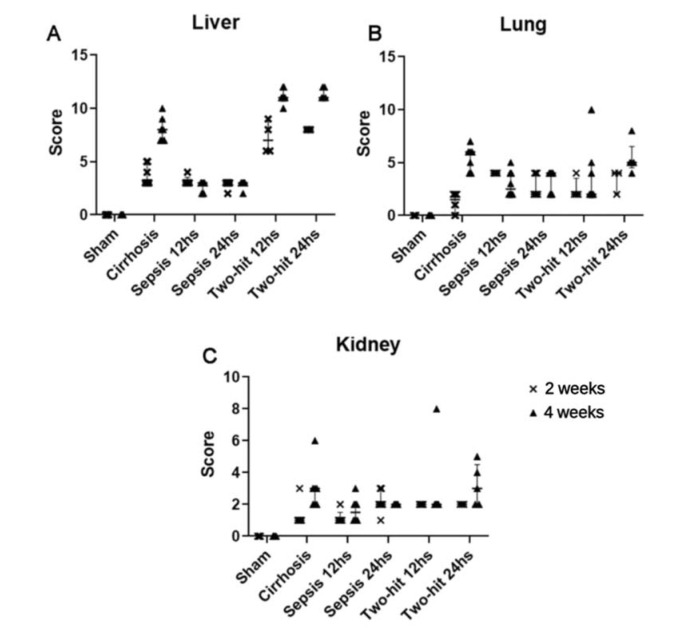
Median score values of histopathological findings (*p<0.05, **p<0.01, ***p<0.001, ****p<0.0001 for comparisons against the sham group).

The main histopathological findings are shown in Figure 3, and the scores are shown in Figure 4. In the liver, the BDL+CLP groups presented significant differences compared to the sham groups, as well as to the respective septic rat groups (*p*<0.001 for 2-to-2 comparisons). The variables of greatest statistical differences were the presence of exudate, followed by ductular proliferation, collagen deposition, and vascular congestion.

In pulmonary histology, there were no significant differences shared between the BDL+CLP groups, except for the 24-hour BDL+CLP group at week 4 that showed significant difference (*p*<0.05) compared to the cirrhotic group at week 2. In addition, purely cirrhotic groups showed significant differences between them. The variables with the greatest statistical difference were alveolar space reduction and congestion with discrete thickening of the pulmonary septa.

Finally, regarding renal findings, there were, again, no significant statistical differences shared between the BDL+CLP groups, except for the 24-hour BDL+CLP group at week 4 that had a significant difference (*p*<0.05) in relation to the cirrhotic group at week 2. In addition, purely cirrhotic groups also showed a significant difference between them at weeks 2 and 4. The variables of greatest statistical difference were presence of edema and dilation with protein debris.

### Clinical scores (MSS)

**Figure 5:**
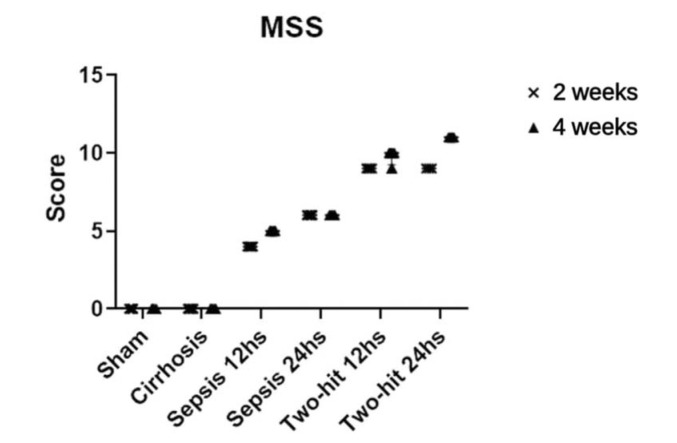
Median score values of MSS (**p<0.01, ***p<0.001, ****p<0.0001 for comparisons with the sham group.

Clinical scores were homogeneous among individuals from the same group (Figure 5). The differences were mainly significant between the BDL+CLP and the sham groups. In addition, at week 2, cirrhosis differed significantly from 12-hour BDL+CLP (*p*=0.001) and 24-hour BDL+CLP (*p*=0.001); at 4 weeks, cirrhosis also differed significantly from 12-hour (*p*<0.0001) and 24-hour BDL+CLP (*p*<0.001). The principal distinctive variables were appearance, eye involvement, activity, response to stimuli, and state of consciousness.

### Correlations

The Spearman rank correlation coefficient (Spearman's rho) was used for the correlation analyses. The correlation of interleukins with MSS was moderate-to-strong, with the highest correlation demonstrated between IL-10 and MSS (Spearman's rho=0.64, *p*<0.0001), followed by IL-1 beta (Spearman's rho=0.57, *p*<0.0001) and TNF-alpha (Spearman's rho=0.56, *p*<0.0001). In addition, the correlations between the following were considered strong-to-very strong: IL-10 and IL-1 beta, TNF-alpha and IL-1 beta and TNF-alpha and IL-10.

On the correlation of histopathological findings with MSS, those of the liver and kidney presented a strong correlation with MSS (Spearman's rho = 0.65 and 0.66, respectively, *p*<0.0001 for both), while the lung presented moderate correlation (Spearman's rho = 0.5, *p*<0.0001). No significant correlations were observed between interleukins and histopathology, except for the relationship between IL-10 and lung.

Correlation between mortality rate and MSS were significant (Spearman's rho=0.9781; *p*<0.0001).

## DISCUSSION

In the current investigation, we focused on a growing animal experimental model to study sepsis and cirrhosis due to the known physiological differences between children and adults in both situations. Young cirrhotic animals present with a less intense response of ductular proliferation and inflammatory infiltration in comparison to adults, despite increased portal and periportal fibrosis (4). Regarding sepsis, there are several peculiarities in children. In neonates, the increased pulmonary resistance and low pulmonary blood flow can result in hypoxemia, which can be sometimes potentiated by acidosis. These phenomena can result in cardiac failure and may indicate treatment with inhaled nitric oxide, oxygen, and phosphodiesterase III inhibitors, which is different from adults in whom these therapies may worsen hypotension and exacerbate multiple organ failure. Another difference is that adults with sepsis generally present with ‘‘warm’’ shock due to peripheral vasodilatation, while 50% of children present with ‘‘cold’’ shock, characterized by peripheral vasoconstriction and limited cardiac reserve. In general, increased heart rate in children with sepsis has no hemodynamic benefits as seen in adults. Their resting heart rates are high in comparison to adults and tachycardia may not allow for increased diastolic filling (9).

Isolated models have been cited in several experimental works and are very practical, including young animal models (10). BDL has already been used before in young animal models with satisfactory results to elucidate cirrhosis pathophysiology (11-15), the same goes with CLP in different age groups (15-19). Also, our previous experiments have verified that BDL promotes intense alterations in liver morphology from the 3^rd^ day until the 4^th^ week, which was the reason why these time periods were chosen in our studies (4-6). In the current work, the BDL+CLP model technique in young rats was feasible and of rapid execution. Regarding mortality, animals subjected to a greater number of procedures, due to the number of interventions and the accumulation of comorbidities, were expected to have a higher mortality rate as seen in previous works (20,21). This seems to correspond with what is observed in clinical practice, wherein individuals with cirrhosis in the surgical context, and concurrent infectious complications tend to present with higher mortality (22).

Results of BDL experimental models confirmed that this is a good surgical model for cirrhosis, owing to simplicity of the technique and visibility of results of the cirrhotic picture. Regarding CLP, previous studies indicated a lower aggressiveness with a single 21 G needle hole, despite being a constant source of bacteria, necrotic tissue and their inflammatory mediators (such as the low concentrations of stool solutions injected into animal models (23-25). Aggressiveness would be slightly higher only in animals with prior comorbidities, as with the BDL+CLP model of the present study.

Furthermore, it has already been shown that very young animals (especially during and after breastfeeding) in septic conditions present with higher levels of inflammatory biomarkers, especially IL-6 and TNF-alpha; which was the reason these interleukins were chosen (26,27). As expected, a fast and fleeting pattern was displayed in this experiment. In general, after 12 hours of CLP, the concentrations, mainly those of IL-1 beta, would initially increase and decrease with the passage of hours, as confirmed in the literature (28,29). No clear specific distinctions in pattern of inflammation were observed between the groups. This was probably due to the unrestricted stratification of infection severity (which was not the focus of the current investigation) and the utilization of different techniques in other septic models (30,31).

In the pathology results evidenced by the histological slides, a pattern of cirrhosis and of sepsis, both macro and microscopically, were observed (Figure 3). The most affected organs were the liver, followed by the kidney and lung, by varying degrees of fibrosis and inflammation.

The kidneys and lungs presented with important and different degrees of necrosis and inflammation (with signs of thickening and congestion) according to the type of injury. These findings were mildly representative of hepatic insufficiency and cirrhosis associated with hepatorenal and hepatopulmonary syndromes that occur in patients with cirrhosis (32,33).

In the slides analyzed, the BDL+CLP septic models showed enhanced inflammatory aspects of the cirrhotic scenario, with greater vascular congestion and ductular proliferation. However, in terms of polymorphonuclear cell and lymphocyte infiltration, their presence, especially in the intraparenchymal region, has so far been scarce. Finally, the heart histological slides did not present significant alterations in any group. Scores computed and analyzed were in line with those presented in the literature and applied to other studies (34,35).

It was observed that clinical scores had a positive correlation with mortality and pathological findings. The comparisons of MSS values demonstrated a progressive increase from the first septic group (12 hours) (Figure 5). Besides, it was noted that groups subjected to the same intervention for the same period, but with different hours of sepsis, had different scores, as observed in the 24-hour groups that had almost twice the mortality rate in comparison with the other groups.

Analysis by means of correlation may help in achieving an integrated interpretation of the different data. In fact, higher levels of serial cytokines were observed in animals with more evident clinical findings of sepsis. In the same way, histological damage of the studied organs (mainly the liver and kidney), as well as mortality rates, were proportional to clinical manifestations of sepsis. Considering that MSS values were higher in the BDL+CLP groups, we can conclude that cirrhosis, in young organisms, leads to a more intense response to infection, and this is expressed by higher levels of cytokines and more intense histological damage in target organs.

Thus, this study has shown to be a good design to simulate septic peritonitis in models with comorbidities (cirrhotic, in this case). However, its application is more restricted to mild or early conditions, with difficult clinical staging within the disease severity spectrum: intense repercussions locally (purulent exudate and vascular congestion), but with systemic repercussions still in progression. This corroborates with the challenge of murine immunity being more &quot;resistant&quot; compared to humans (25,36). Also, as stated before, perhaps a longer observation period with more fragmented times would improve the quality of the models.

## CONCLUSION

The experimental model of growing animals described herein is reproducible, feasible and may be utilized in investigation studies of liver cirrhosis and infection.

## AUTHOR CONTRIBUTIONS

Moraes PAD, Tannuri ACA and Rios LM designed and conducted the experiments, performed the data analysis, and drafted the manuscript. Gonçalves JO and Serafini S were responsible for laboratory studies. Paes VR performed the histopathological interpretations. Tannuri U and Tannuri ACA provided critical revisions of the manuscript for important intellectual content. All authors read and approved the final version of the manuscript.

## Figures and Tables

**Table 1:** Details of histopathological analysis.

Liver
1) Liver enlargement 2) Liver with purulent content 3) Ductular proliferation 4) Collagen in the porta space 5) Inflammatory portal infiltrate 6) Parenchymal infiltrate 7) Vascular congestion

Heart
1) Ischemia / Necrosis 2) Inflammatory Parenchymal Infiltrate 3) Areas of fibrosis 4) Vascular Congestion

Lung
1) Alveolar hemorrhage 2) Airway thickening 3) Septal thickening 4) Inflammatory infiltrate 5) Hyaline membrane 6) Vascular congestion

Kidney
1) Edema 2) Interstitial inflammatory infiltrate 3) Tubular tissue fluctuation and necrosis 4) Tubular dilatation with protein debris

**Table 2:** Murine sepsis score to assess the severity of disease in the experimental groups.

Variable	Score and Description
Appearance	0- Coat is smooth 1- Patches of hair piloerected 2- Majority of back is piloerected 3- Piloerection may or may not be present, mouse appears “puffy” 4- Piloerection may or may not be present, mouse appears emaciated
Level of consciousness	0- Mouse is active 1- Mouse is active but avoids standing upright 2- Mouse activity is noticeably slowed. The mouse is still ambulant. 3- Activity is impaired. Mouse only moves when provoked, movements have a tremor 4- Activity severely impaired. Mouse remains stationary when provoked, with possible tremor
Activity	0- Normal amount of activity. Mouse is any of: eating, drinking, climbing, running, fighting 1- Slightly suppressed activity. Mouse is moving around bottom of cage 2- Suppressed activity. Mouse is stationary with occasional investigative movements 3- No activity. Mouse is stationary 4- No activity. Mouse experiencing tremors, particularly in the hind legs
Response to stimulus	0- Mouse responds immediately to auditory stimulus or touch 1- Slow or no response to auditory stimulus; strong response to touch (moves to escape) 2- No response to auditory stimulus; moderate response to touch (moves a few steps) 3- No response to auditory stimulus; mild response to touch (no locomotion) 4- No response to auditory stimulus. Little or no response to touch. Cannot right itself if pushed over
Eyes	0- Open 1- Eyes not fully open, possibly with secretions 2- Eyes at least half closed, possibly with secretions 3- Eyes half closed or more, possibly with secretions 4- Eyes closed or milky
Respiration rate	0- Normal, rapid mouse respiration 1- Slightly decreased respiration (rate not quantifiable by eye) 2- Moderately reduced respiration (rate at the upper range of quantifying by eye) 3- Severely reduced respiration (rate easily countable by eye, 0.5 s between breaths) 4- Extremely reduced respiration (>1 s between breaths)
Respiration Quality	0- Normal 1- Brief periods of labored breathing 2- Labored, no gasping 3- Labored with intermittent gasps 4- Gasping

**Table 3:** Survival rate of animals in the different groups.

Group (number of animals)	Survival rate (%)
Sham 2ws (8)	100
Sham 4ws (8)	100
BDL 2ws (8)	100
BDL 4ws (8)	100
Sepsis 2ws 12hs (8)	87.5
Sepsis 4ws12hs (13)	92.3
Sepsis 2ws 24hs (11)	63.6
Sepsis 4ws 24hs (11)	81.8
Two-hit 2ws 12hs (12)	75.0
Two-hit 4ws 12hs (8)	62.5
Two-hit 2ws 24hs (10)	60.0
Two-hit 4ws 24hs (13)	61.5
